# Quality of life in urologic cancer patients: importance of and satisfaction with specific quality of life domains

**DOI:** 10.1007/s11136-021-02954-7

**Published:** 2021-07-18

**Authors:** Katja Leuteritz, Diana Richter, Anja Mehnert-Theuerkauf, Jens-Uwe Stolzenburg, Andreas Hinz

**Affiliations:** 1 Department of Medical Psychology and Medical Sociology, University of Leipzig, 04103 Leipzig, Germany; 2 Department of Urology, University of Leipzig, Leipzig, Germany

**Keywords:** Quality of life, Urology, Prostate cancer, Satisfaction, Importance, Health

## Abstract

**Purpose:**

Quality of life (QoL) has been the subject of increasing interest in oncology. Most examinations of QoL have focused on health-related QoL, while other factors often remain unconsidered. Moreover, QoL questionnaires implicitly assume that the subjective importance of the various QoL domains is identical from one patient to the next. The aim of this study was to analyze QoL in a broader sense, considering the subjective importance of the QoL components.

**Methods:**

A sample of 173 male urologic patients was surveyed twice: once while hospitalized (t1) and once again 3 months later (t2). Patients completed the Questions on Life Satisfaction questionnaire (FLZ-M), which includes satisfaction and importance ratings for eight dimensions of QoL. A control group was taken from the general population (*n* = 477).

**Results:**

Health was the most important QoL dimension for both the patient and the general population groups. While satisfaction with health was low in the patient group, the satisfaction ratings of the other seven domains were higher in the patient group than in the general population. The satisfaction with the domain *partnership/sexuality* showed a significant decline from t1 to t2. Multiple regression analyses showed that the domains *health* and *income* contributed most strongly to the global QoL score at t2 in the patient group.

**Conclusion:**

Health is not the only relevant category when assessing QoL in cancer patients; social relationships and finances are pertinent as well. Importance ratings contribute to a better understanding of the relevance of the QoL dimensions for the patients.

## Introduction

Prostate cancer is the most frequently diagnosed cancer among men, with about 1.3 million new cases being diagnosed annually worldwide [1]. The 5-year survival rate for prostate cancer is relatively high, about 83% in Europe [2]. Quality of life (QoL) has become an important quality and decision criterion for cancer patients, medical practitioners, and the healthcare system. Multiple studies have been performed to investigate QoL [3–6] and mental health [7–9] in urologic cancer patients and survivors. Most studies found that, compared with other cancer types, QoL among prostate cancer patients was relatively good [10, 11]. When compared with controls from the general population, prostate cancer survivors often rate global QoL dimensions similarly [12–14]. However, when specific symptoms are considered such as urinary incontinence, bowel or rectal function, or sexual function, prostate cancer patients report significant detriments [15, 16].

Studies on QoL among cancer patients have historically focused mainly on health-related QoL [3, 17]. However, QoL is a broader concept that also includes factors such as professional life, family life, leisure activities, and finances. Factors like these need to be considered as well when evaluating cancer patients’ QoL and developing supportive services.

While QoL assessment instruments generally cover several domains of well-being, they do not consider the subjective importance of these domains to the individual respondents. It is implicitly assumed that each dimension has roughly the same meaning for all participants. This, however, is not necessarily the case. It is known that subjectively important QoL domains correlate more strongly with overall QoL than unimportant ones do. Therefore, attempts have been made to weight the QoL components with the corresponding importance ratings [18], but weighted results were generally similar to the results of the unweighted calculations [18–20]. However, differing subjective importance of QoL domains is also interesting beyond the aspect of weighting. Which areas of life become more important and which become less important after a cancer diagnosis? Does the importance of health increase?, and does the importance of the other dimensions decrease in the perception of cancer patients? The effects of a cancer diagnosis or treatment on the subjective importance of QoL dimensions are largely unknown.

One instrument that allows to shed light on these issues is the Fragebogen zur Lebenszufriedenheit (Questionnaire on Life Satisfaction) FLZ-M [21] since this questionnaire also addresses the subjective importance of QoL dimensions. A normative study was performed that allows to compare the cancer patients’ importance and satisfaction assessments with those of the general population. Since importance and satisfaction can change over time in cancer patients, we performed a study with two measurement points spaced 3 months apart to investigate changes in these variables.

While one way to assess the importance of a life domain is to ask the participants directly to assess the importance they attribute to it, a second approach is to calculate the associations between the specific life domains and a global assessment of QoL. Direct importance ratings of QoL domains can differ from the associations between the satisfaction with these domains and global QoL. While the domains *health* and *family* received the highest mean importance ratings in a large general population study [22], the contribution of the domain *finances* to the variance explanation of global life satisfaction was higher than the contributions of *health* and *friends*. We intend to test whether such relationships between direct importance ratings and regression coefficients for the prognosis of global QoL can also be found in cancer patients.

The aims of this study were (a) to analyze satisfaction with and the subjective importance of QoL components in urologic cancer patients in comparison with the general population, (b) to investigate changes in QoL in the patient sample, and (c) to investigate the degree to which the QoL domains predict overall QoL using regression analyses.

## Methods

### Patient sample

Patients were enrolled between June 2015 and February 2017. Men with urologic cancer receiving treatment in a German university hospital were eligible. Study inclusion criteria were histologically proven urologic carcinoma, age at diagnosis ≥ 18 years, and being able to read and understand the German language. The patients were surveyed at two time points: once during their hospital stay (t1) and again 3 months following hospital release (t2). Most of the patients completed the t1 questionnaire about two days before hospital discharge. For the t2 measurement, the patients received a letter that included the t2 questionnaire with the same instruments and a pre‐stamped envelope. Patients who did not respond to the t2 letter were given one reminder. The study received research ethics committee approval from the ethics board of the Medical Faculty of the University of Leipzig (ref. no. 287-13-07102013). Informed consent was obtained from all of the participants prior to their inclusion in the study.

### General population sample

To compare the results of the cancer patients with those of the general population, we used general population data based on a representative sample (*n* = 5036) of the general German population [23]. It was a cross-sectional study without retesting. The mean age of the cancer patient sample was 63.0 years (SD = 9.5). We selected a subsample of males from the general population sample with a comparable mean age of 62.4 years (SD = 12.9) and a similar distribution of the education levels according to Table 1, resulting in a subsample of 477 males. This general population study was also approved by the ethics board of the Medical Faculty of the University of Leipzig (ref. no. 092-12-05032012).

**Table 1:** Sociodemographic and medical characteristics of the sample (*n* = 173)

	N	%
Age (Mean, SD) in years	M = 63.0, SD = 9.5	
19–39 years	5	2.9
40–59 years	49	28.3
60–69 years	76	43.9
≥ 70 years	43	24.9
Relationship status*		
Living alone	20	11.6
Living with partner	152	87.9
Education		
< 10 years	19	11.0
11–12 years	65	37.6
≥ 12 years	89	51.4
Tumor site		
Penis	1	0.6
Prostate	150	86.7
Testis	6	3.5
Kidney	7	4.0
Bladder	8	4.6
Ureter	1	0.6
Tumor stage*		
0	2	1.2
I	15	8.7
II	110	63.6
III	30	17.3
IV	15	8.7
Time since diagnosis		
< 1 week	158	91.3
1 week to < 1 month	7	4.0
≥ 1 month	8	4.6
Medical treatment		
Surgery	162	93.6
Chemotherapy	16	9.2
Radiotherapy	4	2.3
Hormone therapy	8	4.6

^*^Missing data not reported; *M* mean; *SD* standard deviation

### Instruments

Several sociodemographic characteristics (age, relationship status, education) were assessed via self-declaration, and medical characteristics (cancer diagnosis, UICC stage, treatment, time since diagnosis) were taken from the clinical patient records.

#### Questions on life satisfaction (FLZ-M)

QoL was measured with the Questions on Life Satisfaction (FLZ-M) [21] at t1 and at t2. This instrument evaluates the respondent’s subjective QoL. It has been used in several medical fields such as cancer [24–26], orthopedics [27], and psychiatry [28]. The questionnaire covers eight areas of life that are assumed to be relevant for most people in the Western world: (1) *Friends/Acquaintances*, (2) *Leisure activities/Hobbies*, (3) *Health*, (4) *Income/Financial security*, (5) *Work/Profession*, (6) *Housing situation*, (7) *Family life/Children*, and (8) *Partnership/Sexuality*. The respondents are asked to rate how important each of these areas is and how satisfied they have been with that area over the previous 4 weeks. The subjects rate the importance of and their satisfaction with the domains on a 5-point Likert scale (range 1–5), whereby higher scores indicate higher levels of importance and satisfaction. The reliability of this instrument is good with Cronbach’s alpha coefficients of 0.81 [21] and 0.80 [23] in general population samples. Convergent and discriminant validity were proved, and the 1-week test–retest reliability of the total score was 0.87 [21]. Norm values are available [23]. In a sample of cancer survivors, Cronbach’s alpha was 0.72 [29], and correlations with anxiety, depression, post-traumatic growth, resilience, and QoL [25, 29] supported the validity of the instrument in oncological samples.

#### EORTC QLQ-C30

The participants also completed the EORTC QLQ-C30 [30]. This QoL questionnaire consists of 30 items and includes five functioning scales, nine symptom scales, and a two-item global health/QoL scale. One of the two items of this subscale asks the respondents to rate their QoL on a scale of 1 = very poor to 7 = excellent. We preferred using this single-item over the two-item global health/QoL scale because that scale has a focus on the health domain, an area we did not want to favor over the others in this study.

### Statistical analysis

Group differences between patients and the general population were tested using *t* tests for unpaired groups; and mean score changes in the patient group were tested with paired *t* tests.

Effect sizes *d* were calculated according to Cohen [31] to indicate the mean score difference between the groups, adjusted for the pooled standard deviation. The association between the satisfaction ratings for the QoL domains (at t1) and global QoL (EORTC QLQ-30 item of global QoL at t2) was analyzed with multiple regression analyses. Two models were calculated: Model M1 included the covariables age, education, and tumor stage, and Model M2 used these three covariables in addition to the baseline value (t1) of QoL as the independent variables. All analyses used Method = Enter. Statistics were performed with SPSS version 24.

## Results

### Sample characteristics

A total of 212 patients treated for urogenital cancer at a German university hospital were eligible for this study. Of these, 197 (response rate: 93%) completed the t1 questionnaires and received the t2 questionnaire 3 months later. With a dropout/failure rate between baseline and follow-up of 12% (24 patients), a total of 173 complete data sets (88% of the t1 respondents) could be analyzed after the 3-month follow-up measurement. On average, the patients were aged 63.0 years (SD = 9.5) at t1 (Table 1). Most patients were surveyed within a week of being diagnosed. The most frequent diagnosis was prostate cancer (*n* = 150; 86.7%). Further characteristics of the sample are given in Table 1.

The selected sample of the general population was aged 62.4 years (SD = 12.9). Of them, 66 (13.8%) attended school < 10 years, 175 (36.7%) for 10–11 years, and 236 (49.5%) for at least 12 years.

### Importance of and satisfaction with life domains for patients and the general population

Importance and satisfaction mean scores of the life domains are given in Tables 2 and 3. Concerning the **importance** of the domains, the patients rated *health* and *family life/children* most highly (Table 2). There was a slight but statistically significant reduction from t1 to t2 in the importance rating of *partnership/sexuality* (*d* = − 0.177; *p* = 0.018). When compared with the general population, the patients (t1) attributed significantly more importance to four of the eight domains (*friends, leisure time, family life*, and *partnership*) and less importance to *income*. The importance of *health* was rated nearly equally by both samples.

**Table 2:** **Importance** ratings. Mean scores and effect sizes for patients (*n* = 173) and the general population (*n* = 477)

	Mean scores and SDs						Differences					
	Patients t1		Patients t2		G.P		Patients t1−G.P		Patients t2−G.P		Patients t2−Patients t1	
	M	(SD)	M	(SD)	M	(SD)	Effect size *d*	*p*	Effect size *d*	*p*	Effect size *d*	*p*
Friends	3.76	(0.82)	3.71	(0.79)	3.54	(0.83)	**0.27**	**0.003**	**0.21**	**0.018**	− 0.06	0.390
Leisure time	3.71	(0.85)	3.66	(0.70)	3.45	(0.87)	**0.30**	** < 0.001**	**0.26**	**0.002**	− 0.05	0.449
Health	4.49	(0.59)	4.51	(0.58)	4.58	(0.60)	− 0.17	0.060	− 0.12	0.147	0.03	0.624
Income	3.68	(0.76)	3.79	(0.81)	4.13	(0.67)	− **0.65**	** < 0.001**	− **0.47**	** < 0.001**	0.14	0.123
Work	3.70	(0.87)	3.73	(0.86)	3.61	(1.26)	0.08	0.100	0.10	0..171	0.05	0.630
Housing	3.88	(0.78)	3.84	(0.87)	3.97	(0.73)	− 0.12	0.311	− 0.16	0.100	− 0.04	0.645
Family life	4.35	(0.80)	4.24	(0.83)	4.01	(0.99)	**0.36**	** < 0.001**	**0.24**	**0.006**	− 0.13	0.069
Partnership	3.98	(0.88)	3.82	(0.81)	3.76	(1.02)	**0.22**	**0.009**	0.06	0.453	− **0.18**	**0.018**

*G.P.* general population; *M* mean; *SD* standard deviation. Bold type indicates statistical significance with *p* < 0.05

**Table 3:** **Satisfaction** ratings. Mean scores and effect sizes for patients (*n* = 173) and the general population (*n* = 477)

	Mean scores and SDs						Differences					
	Patients t1		Patients t2		G.P		Patients t1−G.P		Patients t2−G.P		Patients t2−Patients t1	
	M	(SD)	M	(SD)	M	(SD)	Effect size *d*	*p*	Effect size *d*	*p*	Effect size *d*	*p*
Friends	4.06	(0.83)	3.97	(0.86)	3.86	(0.76)	**0.26**	**0.004**	0.13	0.146	− 0.11	0.107
Leisure time	3.93	(0.93)	3.64	(0.92)	3.77	(0.81)	**0.19**	**0.031**	− 0.16	0.093	− **0.31**	** < 0.001**
Health	3.08	(1.20)	3.27	(1.10)	3.58	(0.90)	− **0.50**	** < 0.001**	− **0.33**	**0.001**	**0.17**	**0.022**
Income	3.80	(0.91)	3.74	(0.97)	3.66	(0.84)	0.17	0.055	0.09	0.283	− 0.06	0.306
Work	3.82	(0.97)	3.58	(1.06)	3.59	(1.02)	**0.23**	**0.011**	− 0.01	0.954	− **0.23**	**0.001**
Housing	4.38	(0.77)	4.33	(0.81)	4.11	(0.76)	**0.35**	** < 0.001**	**0.28**	**0.002**	− 0.06	0.273
Family life	4.30	(0.96)	4.14	(0.98)	4.02	(0.86)	**0.31**	** < 0.001**	0.14	0.149	− **0.16**	**0.012**
Partnership	3.76	(1.12)	3.31	(1.07)	3.72	(1.05)	0.03	0.753	− **0.39**	** < 0.001**	− **0.41**	** < 0.001**

*G.P.* general population; *M* mean; *SD* standard deviation. Bold type indicates statistical significance with *p* < 0.05

The life domains the patients reported having the highest satisfaction (Table 3) with were *housing* and *family life*, while *health* and *partnership* received the lowest scores. Significant changes in satisfaction from t1 to t2 were found in five of the eight domains. Satisfaction increased significantly in the *health* dimension (*d* = 0.168; *p* = 0.022) and decreased significantly in four domains: *partnership* (*d* = − 0.412), *leisure time* (*d* = − 0.314), *work* (*d* = − 0.235), and *family life* (*d* = − 0.165). When compared with the general population, the patients’ satisfaction ratings (at t1) were higher in all of the dimensions except health.

### Factors associated with global life satisfaction

The potential of the eight QoL dimensions for predicting general QoL at t2 is analyzed in Table 4. Two models are considered, separately for each dimension. Model 1 includes the satisfaction rating of the domain at t1, together with three covariables: age, education, and tumor stage, as predictors of global QoL at t2. In model 2, the baseline score of QoL is added as a predictor. Concerning model 1, seven of the eight domains contribute significantly to QoL at t2, the only exception is the domain *partnership*. The highest *β* score was found for the dimension *health* (*β* = 0.418), followed by *income* (*β* = 0.386). When the QoL baseline value is included in the regression (model 2), the *β* coefficients of the eight domains become smaller, but still remain statistically significant for five dimensions (*friends, health, income, work*, and *housing*). *Health* has the greatest impact on QoL at t2 (*β* = 0.323) even after controlling for baseline QoL.

**Table 4:** Results of the regression analyses with global QoL at t2 as the dependent variable

	Model	Adj. R^2^	Age		Education		Stage		Domain baseline		QoL baseline	
			*β*	*p*	*β*	*p*	*β*	*p*	*β*	*p*	*β*	*p*
Friends	M1	0.079	**0.170**	**0.023**	0.045	0.520	** − 0.177**	**0.019**	0.**200**	**0.008**		
	M2	0.199	0.091	0.201	0.056	0.423	** − 0.145**	**0.034**	0.**157**	**0.025**	**0.361**	** < 0.001**
Leisure time	M1	0.106	0.120	0.109	0.029	0.700	**0.177**	**0.017**	0.**260**	**0.001**		
	M2	0.189	0.072	0.318	0.042	0.552	** − 0.151**	**0.033**	0.135	0.079	**0.328**	** < 0.001**
Health	M1	0.206	0.067	0.349	0.081	0.252	** − 0.173**	**0.013**	0.**418**	** < 0.001**		
	M2	0.261	0.032	0.642	0.078	0.249	** − 0.151**	**0.025**	0.**323**	** < 0.001**	**0.265**	** < 0.001**
Income	M1	0.179	0.062	0.396	0.022	0.758	** − 0.187**	**0.009**	0.**386**	** < 0.001**		
	M2	0.260	0.016	0.819	0.034	0.619	** − 0.158**	**0.020**	0.**311**	** < 0.001**	**0.306**	** < 0.001**
Work	M1	0.099	0.105	0.171	0.032	0.668	** − 0.174**	**0.020**	0.**252**	**0.001**		
	M2	0.196	0.055	0.449	0.044	0.536	** − 0.147**	**0.037**	0.**158**	**0.035**	**0.337**	** < 0.001**
Housing	M1	0.113	0.120	0.106	0.013	0.862	** − 0.191**	**0.010**	0.**276**	** < 0.001**		
	M2	0.222	0.054	0.444	0.027	0.693	** − 0.157**	**0.024**	0.**223**	**0.002**	**0.346**	** < 0.001**
Family life	M1	0.068	0.132	0.086	0.046	0.545	** − 0.174**	**0.022**	0.**172**	**0.025**		
	M2	0.186	0.066	0.366	0.053	0.453	** − 0.144**	**0.043**	0.113	0.119	**0.362**	** < 0.001**
Partnership	M1	0.045	**0.156**	**0.044**	0.040	0.598	** − 0.188**	**0.014**	0.076	0.322		
	M2	0.175	0.081	0.268	− 0.050	0.488	** − 0.152**	**0.034**	0.035	0.622	**0.376**	** < 0.001**

Domain baseline: t1 score of the respective domain; *M1* Model 1 with the independent variables age, education, stage, and domain baseline; *M2* Model 2 with the independent variables of M1 and the t1 score of global QoL; Bold type indicates statistical significance with *p* < 0.05

Of the covariables, only tumor stage was statistically significant in all of the analyses, while the impact of age and education was small and insignificant in most cases.

## Discussion

While the impact of urologic cancer on health-related QoL has been examined in multiple studies, the aim of the work presented here was to test whether other QoL domains are also affected by the disease and whether the subjective importance ratings of various QoL dimensions differ between urologic cancer patients and the general population. Health is one of the several QoL dimensions included in our analyses; this allows us to investigate the relevance of health in relation to other areas of QoL.

The most relevant QoL dimensions were *health* and *family life*, each of which had mean importance scores above 4 on a scale of 1–5. However, the general population also considers *health* to be the most **important** dimension; there were no significant differences in the *health* importance assessments between the patients and the general population. Other general population studies have also reported health receiving the highest importance ratings [19, 32]. While the patients’ mean importance ratings were higher than those of the general population in four of the seven other dimensions, the other three dimensions showed an opposite trend. This means that the non-health domains do not become less relevant for people after they have been diagnosed with cancer. As such, it is important to consider problems concerning finances, work, and social relationships when studying patient QoL, as these less physical aspects of life appear to be highly relevant for patients as well [33].

Concerning **satisfaction**, it is not surprising that the most relevant difference between the patients and the general population was found for the *health* domain. Nevertheless, the patients’ mean satisfaction rating was 3.08 which is nearly exactly the middle of the 1–5 scale, rather than in the lower half of the scale as one might expect. The patients’ satisfaction ratings were higher than those of the general populations in all of the other domains and in five of the seven cases even with statistically significant differences. This could be a result of a judgment effect: when there are severe detriments in one area (health in this case), the problems in other areas seem to become less relevant. To gain a better understanding of a person’s satisfaction with their health state, it might be useful to consider not only their satisfaction with their health alone but also with their health in relation to their general satisfaction with other areas as well.

During the 3-month period between t1 and t2, satisfaction scores slightly improved in the *health* domain (effect size *d* = 0.17), but became worse in the *partnership* domain, with a large effect size of *d* = − 0.41. The item includes both partnership and sexuality. While in most cases the combination of partnership and sexuality in one dimension makes sense, for prostate cancer patients, these sub-domains can be experienced quite differently. Several patients reported for example that they were highly satisfied with their partnership but very dissatisfied with their sexuality. Since urologic cancer patients often experience urinary and sexual symptoms that do not disappear within the first months after surgery [12, 15, 34, 35], the loss in satisfaction with the combined *partnership/sexuality* dimension is understandable. Partnership and sexuality are areas of life that deserve special attention in the treatment of urologic cancer patients and survivorship care plans [15, 36–39]. A US-American study showed poorer quality of sexual communication and more sexual dissatisfaction after treatment in patients than in the general population [40]. Moreover, patients´ relationship satisfaction, quality of communication about sexuality, and sexual satisfaction were strongly associated with their partner’s satisfaction with the overall treatment outcome [15] and partners´ level of depression and sexual activity [40].

When considering the changes in QoL scores from t1 to t2, one must take into account that they might have been affected by response shift processes, whereby the respondents’ frames of reference changed due to adaptation processes [41–43]. A study with prostate cancer patients [42] tried to quantify this effect and to estimate “true” changes. So-called thentests [44] could be used to further explain such effects and to better understand the real changes.

How do the eight QoL dimensions contribute to global QoL scores at t2? The results of the regression analyses (Model 1) show that all of the dimensions positively contribute to this global score and that the only non-significant dimension is *partnership*. The highest contributions came from the dimensions *health* (*β* = 0.418) and *income* (*β* = 0.386). Even after including the baseline value in the regression analyses (model 2), the domains with the highest *β* values were *health* (*β* = 0.323) and *income* (*β* = 0.311). A general population study [22] found that income was the strongest predictor of general life satisfaction (*r* = 0.59), while health was a weaker predictor (*r* = 0.46) and comparable with the dimensions friends (*r* = 0.45) and job (*r* = 0.47). It would be interesting to compare the associations between health satisfaction and general life satisfaction between patients and the general population in a more systematic way. Our analyses were controlled for age, education, and tumor stage. Therefore, these factors cannot be considered confounders for the effects. The relevance of the domain *income* seems to contradict the low importance ratings of this domain. While the patients declare that income is not so relevant for them, those patients who are satisfied with their income report a higher overall QoL than those who are less satisfied with their income. There is no linear relationship between the direct, explicit importance ratings of the dimensions and the indirect assessments based on associations with global QoL. While both analytical approaches reveal the *health* dimension to be highly relevant, the *income* dimension shows that the results of these two approaches may differ considerably. A similar phenomenon was observed in a general population study [22] where the domains with highest mean importance ratings were not necessarily those with the highest capability for predicting global life satisfaction. This shows that direct assessments of subjective importance must be considered with caution.

Some **limitations** of this study should be mentioned. While multiple studies have investigated health-related QoL in urologic cancer patients, assessments of QoL areas beyond health are rare, and considering the subjective importance of other life domains is a relatively new pursuit. Until now, there are only few applications of the FLZ-M in oncological research, and we could not compare our main findings with results obtained in the scientific literature. The dimension *partnership/sexuality* included two components which, in the case of urological cancer patients, do not form a consistent scale. We showed that direct importance assessments and indirect assessments in terms of *β* coefficients can result in different outcomes. While *health* was relevant in both approaches, the *income* dimension showed contradictory results. We cannot derive conclusions about the best way to infer the subjective relevance; a more stringent comparison between these direct and indirect methods would be a task for future research. Though the response rate of this study was relatively good, it is possible that the proportion of patients with severe problems is underrepresented since the t2 sample included only those study participants who had survived until at least 3 months after t1 and who were willing and able to take part in the t2 assessment. The time interval of 3 months is not sufficient for conclusions about long-term changes in QoL; however, in five of the eight dimensions of QoL, significant changes in the satisfaction ratings could be established. Though we tried to select a control group with a similar distribution of age and education, there may be differences with regard to other aspects such as income we could not control for. We addressed several research questions in this paper, but the data set can also be used for testing other relationships, e.g., the correlations between the importance and the satisfaction ratings, or testing the “domain-importance-as-a-leveler-hypothesis” [32] that postulates a moderating effect of the domain importance on the associations between domain satisfaction and global QoL, or the associations between changes in importance (from t1 to t2) and changes in satisfaction. In our study, we compared the QoL dimension *health* with other dimensions of QoL and did not perform a detailed analysis of health-related QoL in urologic cancer patients. For those purposes, special instruments such as the prostate-specific module EORTC QLQ-PR25 [45] are available, and from our study, we cannot derive conclusions for dealing with QoL problems that are specific for urologic cancer patients. Studies with patients suffering from other cancer entities should be performed to further validate the instrument in oncologic settings and to evaluate the generalizability of the findings of this examination.

In **summary**, the results of this study underline that health is a relevant dimension of QoL but not solely so. The importance of the domain *income/finances* shows that this aspect is also meaningful for understanding cancer patients’ life situation, even if they do not explicitly state that to be the case. The domain *partnership/sexuality* is especially sensitive for urologic cancer patients and should be taken into account in the cancer care setting. Domain importance is meaningful. Even if importance ratings are not necessary for qualifying a weighted global QoL score, they are useful tools for better understanding what is truly relevant for patients [46].

## Data Availability

Data supporting the findings will be made available on reasonable request.
